# Supplementary material for: Detection of genomic regions associated malformations in newborn piglets: a machine-learning approach
Source: PeerJ. 2021 Jul 22;9:e11580. doi: 10.7717/peerj.11580 (PMC8310618; doi:10.7717/peerj.11580)
Supplement: Supplemental Information 1 — (1) PCA (2) Overlap between SNP lists [file peerj-09-11580-s001.docx]

Supplementary for “Detection of genomic regions associated malformations in newborn piglets: a machine-learning approach” by Bakoev et al.

**Supp Fig 1. PCA analysis.** PCA shows the arch and horseshoe effects for Large White pigs. These effects are caused by the unimodal distribution of species along gradients.

Black dots – Landrace pigs. Red dots – Large White pigs.


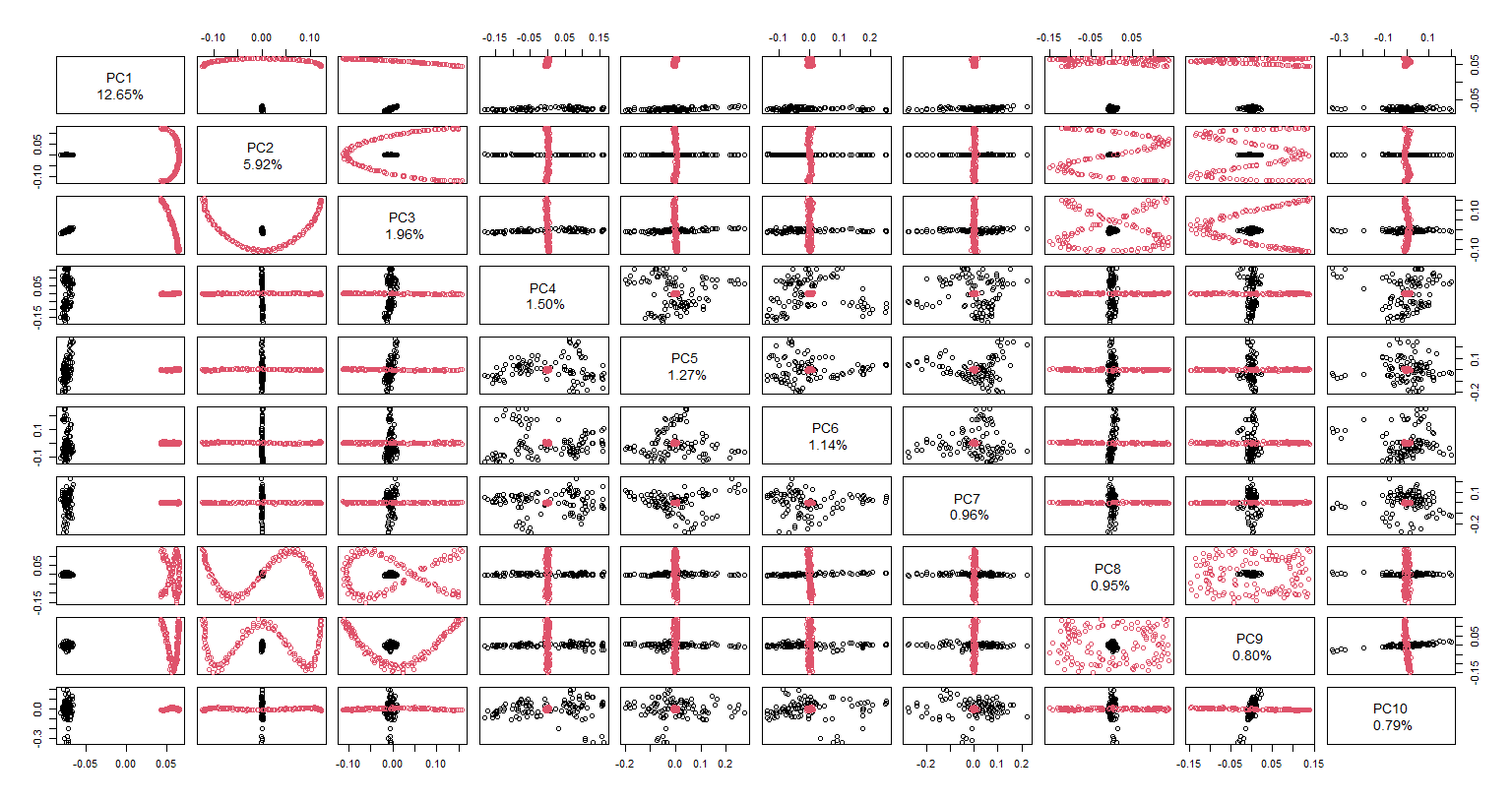


To understand the general trends for gene selection by the two methods, we have lowered the threshold and combined the SNPs identified for both breeds and malformations. We have selected SNPs with p-value<10^-2^ (1595 SNPs, see Supp. Table 1 for details) and determined the effects of these variants using the *Ensembl!* Variant Effect Prediction tool: 70% of the SNPs were intron variants, 7% intergenic, 6% non-coding transcript variants, 5% downstream gene variants, 4% upstream gene variants, 2% synonymous variants, 1% 3-prime UTR variants, and 1% missense variants (Supp. Table 2). Next, we have found human orthologs of the genes containing these high-scoring SNPs and found the traits that these SNPs in genes were associated with, using the EBI GWAS Catalog and determined the most frequent traits. Not surprisingly, we found weight- and body-type-related traits in humans: Height, Metabolite levels, Body mass index, Heel bone mineral density, Obesity-related traits, Type 2 diabetes, HDL cholesterol levels, Waist circumference adjusted for body mass index, Waist-hip ratio, Waist-to-hip ratio adjusted for BMI, Hip circumference adjusted for BMI, Low-density lipoprotein cholesterol levels, LDL cholesterol levels, Waist circumference, Hip circumference, Weight, Appendicular lean mass, Bone mineral density (hip). In the top five most abundant categories, 62% of genes are associated with body size.

For each test we have selected SNPs with p-value<10^-2^ (for a total of 1595 SNPs from all tests, see Supp Table 3 for details) and determined these variants' effects using the *Ensembl* Variant Effect Prediction tool: intron variants: 73%, intergenic variants: 7%, non-coding transcript variant: 6%, downstream gene variant: 5%, upstream gene variant: 5%, synonymous variant: 2%, 3’ UTR variant: 1%, non-coding transcript exon variant: 1%, missense variant: <1% (Supp Table 4). SNP lists from Basic Allelic and Cochran-Armitage trend tests have the largest overlap with the rrBLUP test. The correlation coefficient between the logarithms of SNP Plink and rrBLUP p-values were 0.76 for the splay leg phenotype and 0.62 for other congenital anomalies. Overall, rrBLUP and Plink are in agreement (see Supp Fig 4).

**Supp Fig 2. Comparison of three methods for congenital anomalies phenotype.** Intersection between SNP lists. Blue: GBM, Red; PLINK, Green: rrBLUP.

| 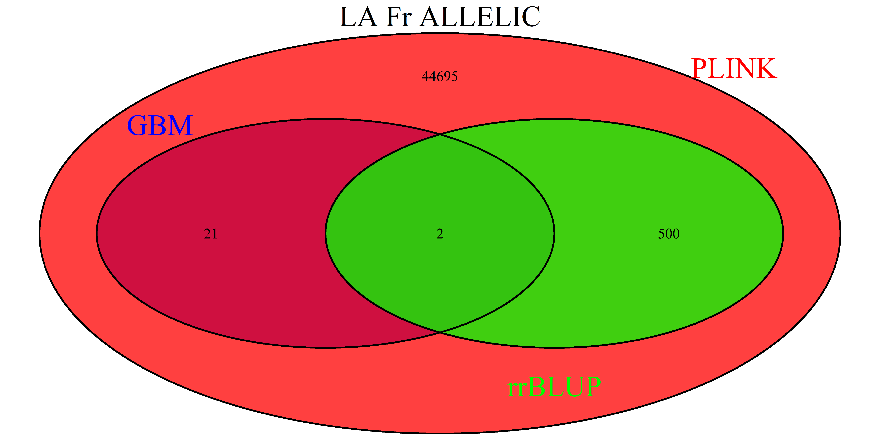 | 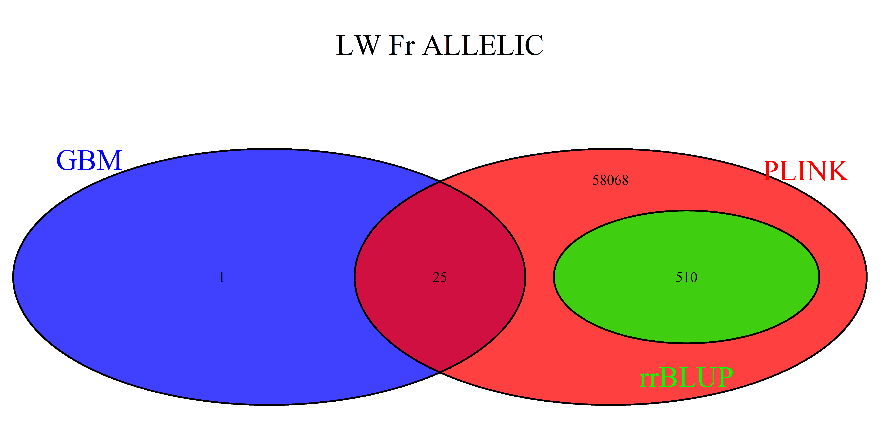 |
| --- | --- |
| 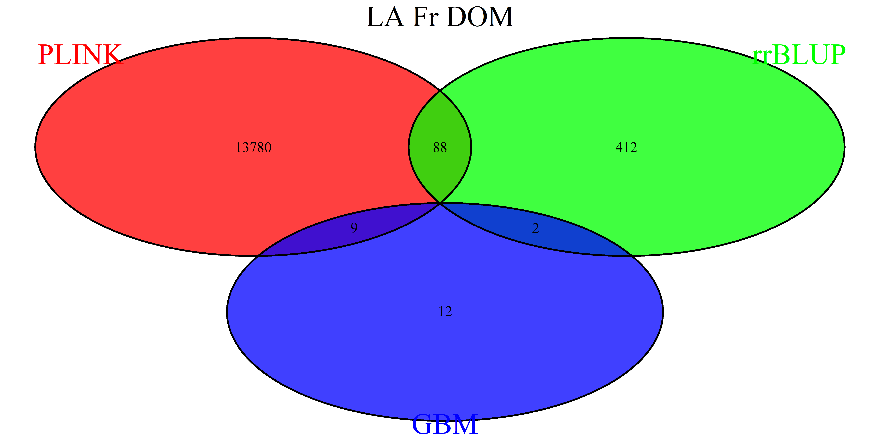 | 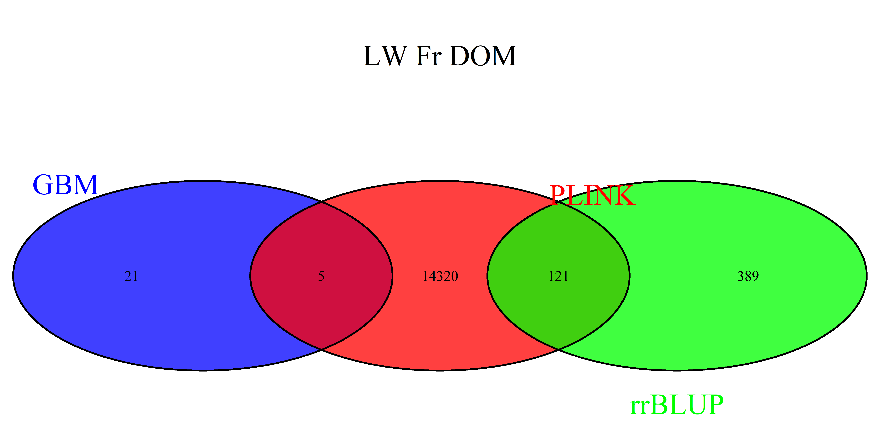 |
| 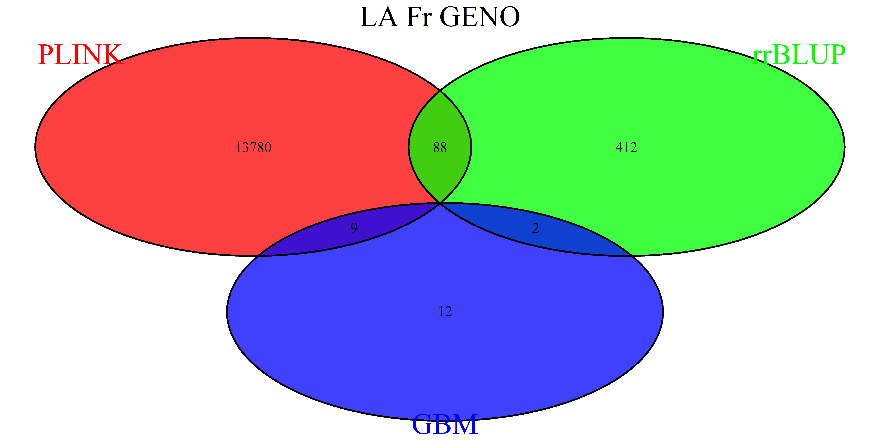 | 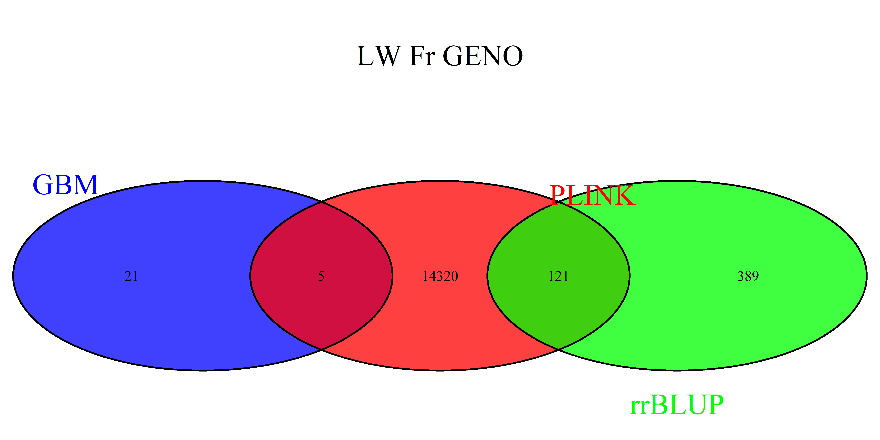 |
| 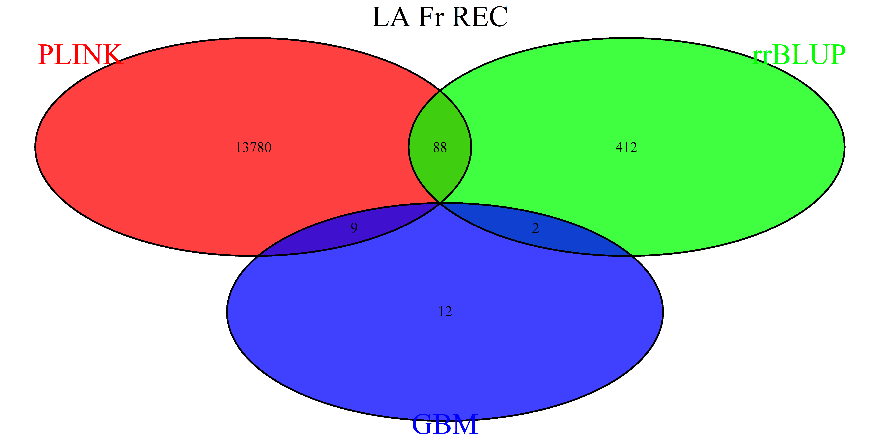 | 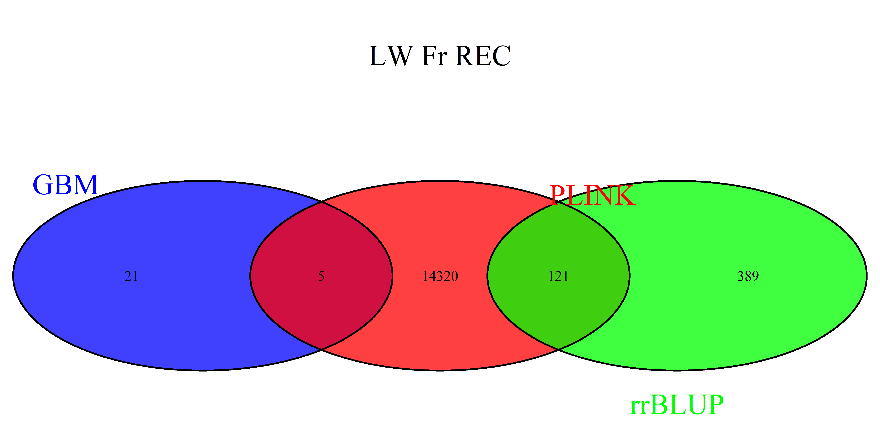 |
| 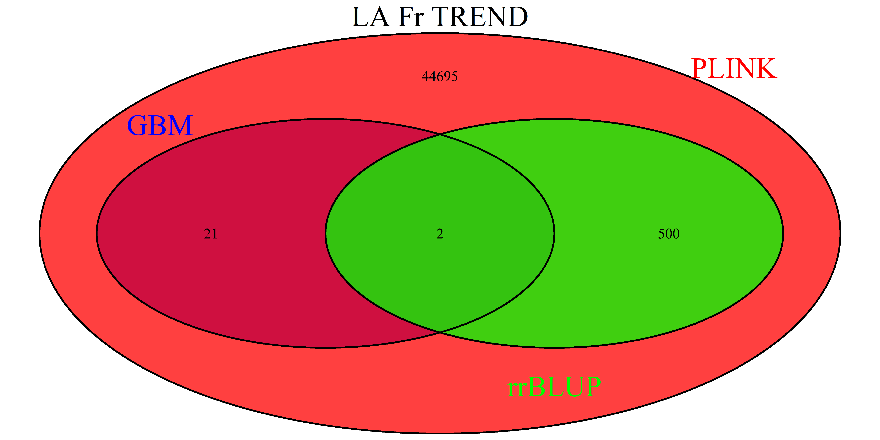 | 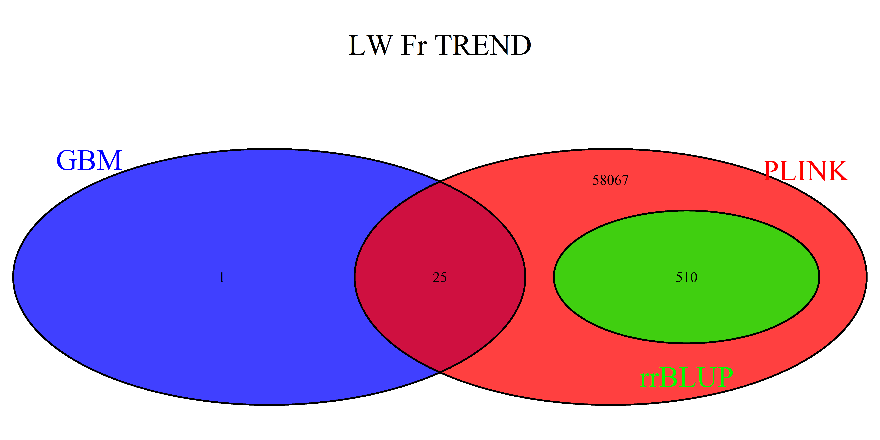 |

| **SNP** | **Location** | **Allele** | **Consequence** | **Existing variant** |
| --- | --- | --- | --- | --- |
| CASI0008977 | [2:107190210-107190210](https://uswest.ensembl.org/Sus_scrofa/Location/View?contigviewbottom=variation_feature_variation%3Dnormal;db=core;r=2:107190160-107190260;tl=OqCodKbrRyXxK2jb-6853557) | G | Intergenic variant | [rs81362278](https://uswest.ensembl.org/Sus_scrofa/Variation/Explore?db=core;tl=OqCodKbrRyXxK2jb-6853557;v=rs81362278) |
| ALGA0021723 | [3:129009157-129009157](https://uswest.ensembl.org/Sus_scrofa/Location/View?contigviewbottom=variation_feature_variation%3Dnormal;db=core;r=3:129009107-129009207;tl=OqCodKbrRyXxK2jb-6853557) | T | Intergenic variant | [rs81378521](https://uswest.ensembl.org/Sus_scrofa/Variation/Explore?db=core;tl=OqCodKbrRyXxK2jb-6853557;v=rs81378521) |

**Supp Fig 3. Comparison of three methods for piglet splay leg phenotype.** The intersection between SNP lists. Blue: GBM, Red; PLINK, Green: rrBLUP.

| 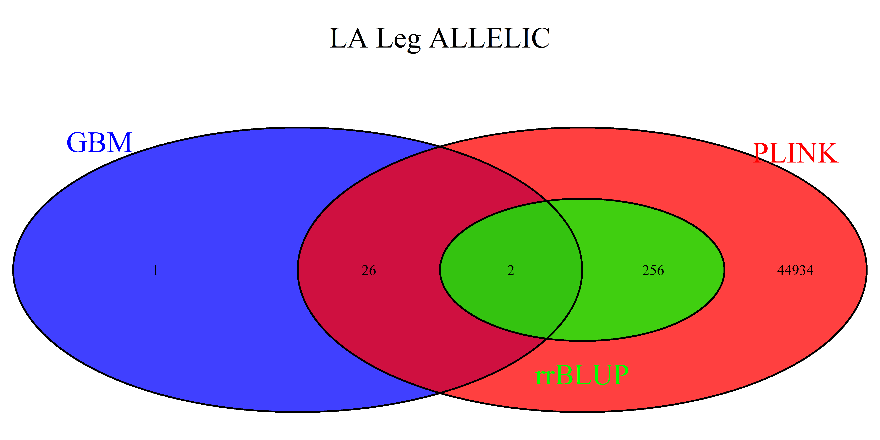 | 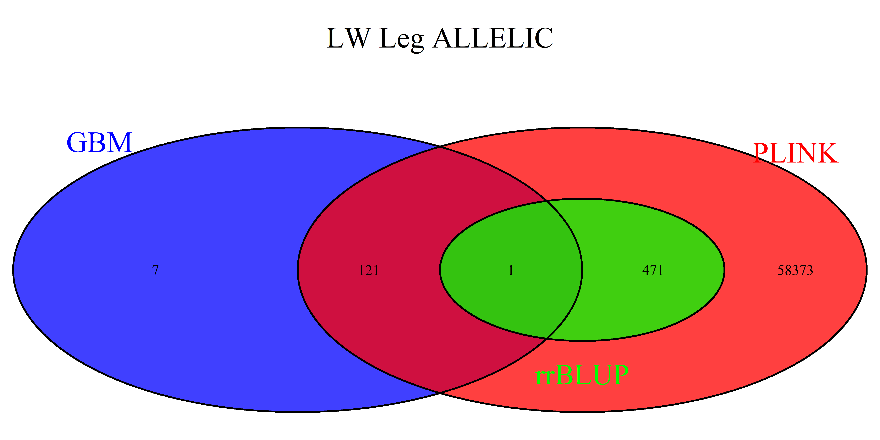 |
| --- | --- |
| 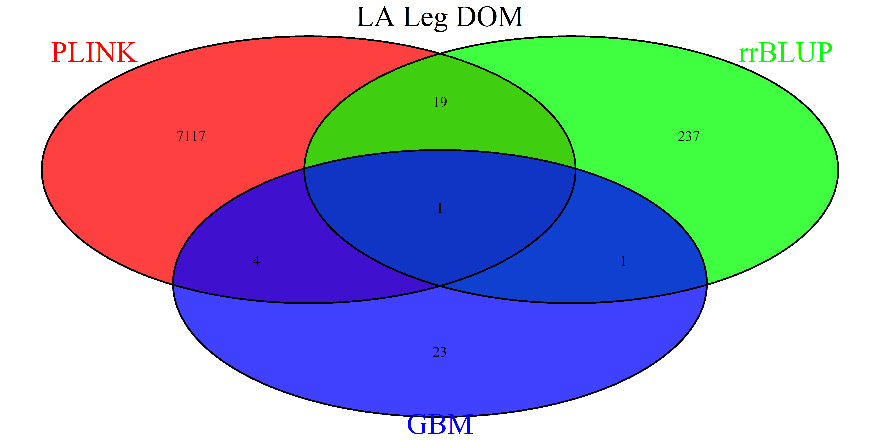 | 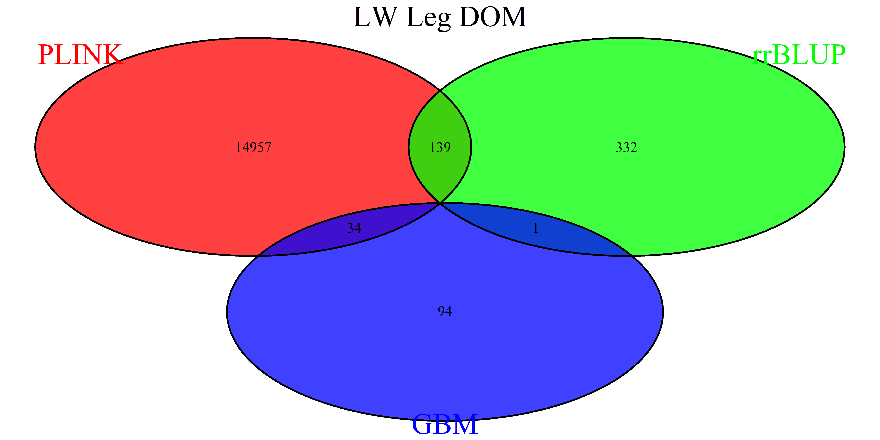 |
| 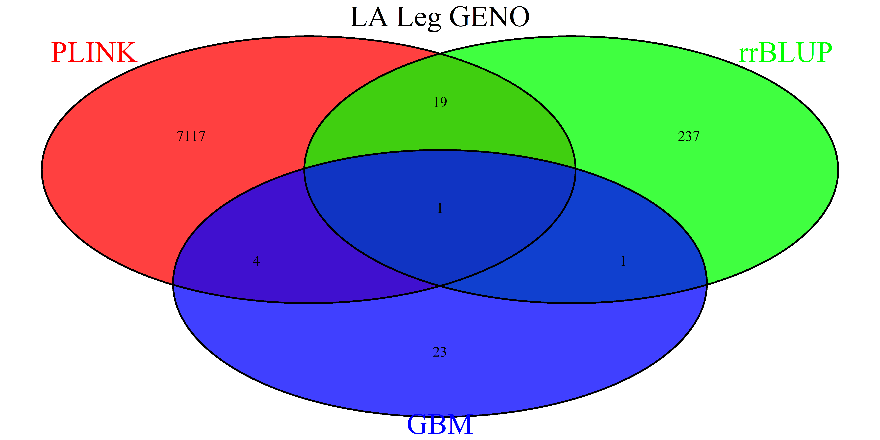 | 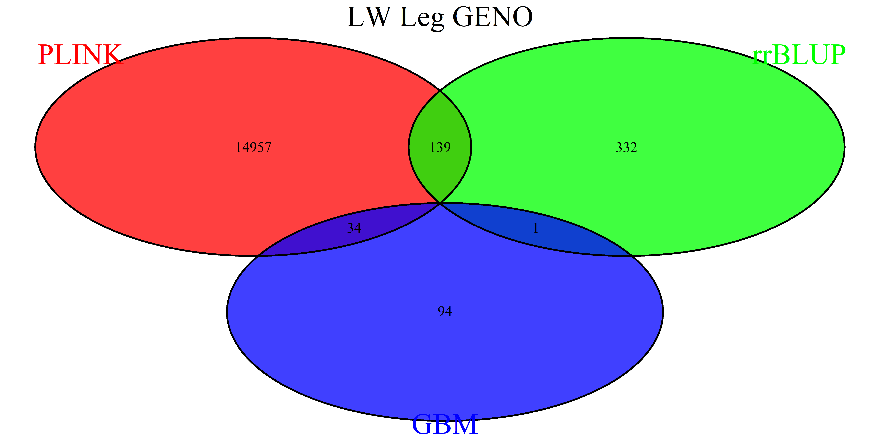 |
| 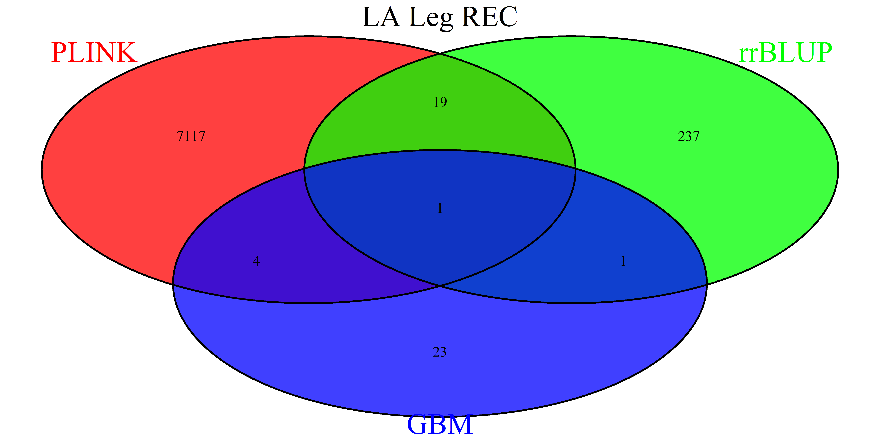 | 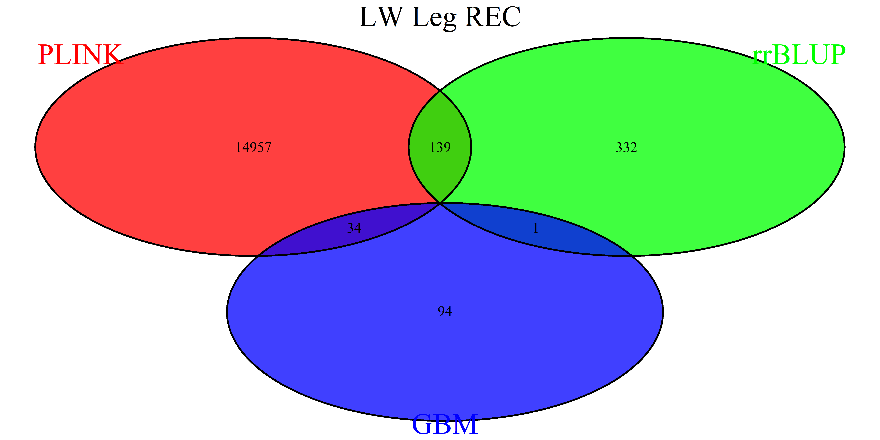 |
| 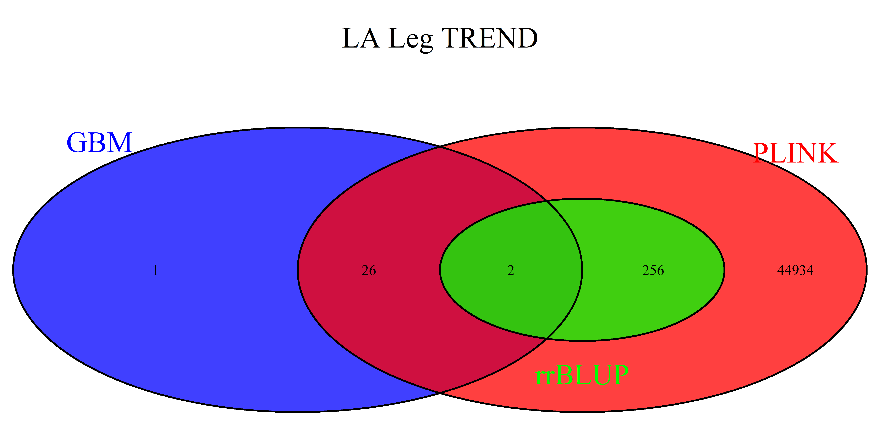 | 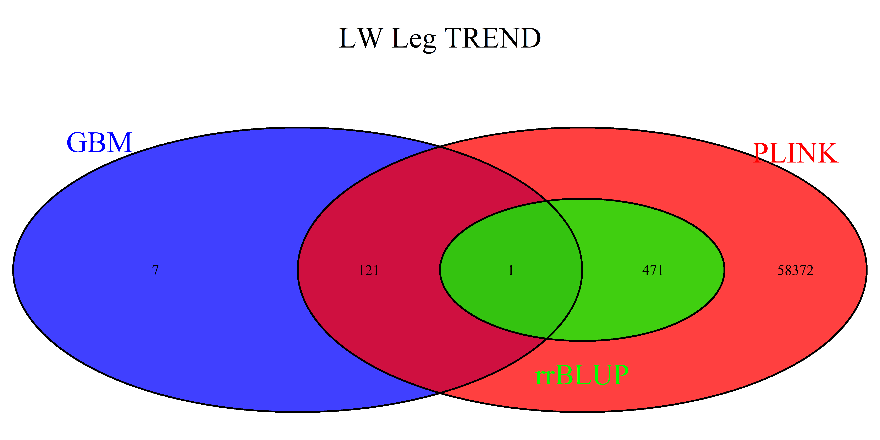 |

| **SNP** | **Location** | **Allele** | **Consequence** | **Symbol** | **Gene** | **Biotype** | **Existing variant** |
| --- | --- | --- | --- | --- | --- | --- | --- |
| MARC0030657 | [1:112210717-112210717](https://uswest.ensembl.org/Sus_scrofa/Location/View?contigviewbottom=variation_feature_variation%3Dnormal;db=core;r=1:112210667-112210767;tl=L04JT1jEsfd9sjP1-6853562) | G | Intron variant,  Non-coding transcript variant | - | [ENSSSCG00000048340](https://uswest.ensembl.org/Sus_scrofa/Gene/Summary?db=core;g=ENSSSCG00000048340;tl=L04JT1jEsfd9sjP1-6853562) | lncRNA | [rs81225364](https://uswest.ensembl.org/Sus_scrofa/Variation/Explore?db=core;tl=L04JT1jEsfd9sjP1-6853562;v=rs81225364) |
| ALGA0075476 | [14:12790416-12790416](https://uswest.ensembl.org/Sus_scrofa/Location/View?contigviewbottom=variation_feature_variation%3Dnormal;db=core;r=14:12790366-12790466;tl=L04JT1jEsfd9sjP1-6853562) | A | Intron variant | HMBOX1 | [ENSSSCG00000009682](https://uswest.ensembl.org/Sus_scrofa/Gene/Summary?db=core;g=ENSSSCG00000009682;tl=L04JT1jEsfd9sjP1-6853562) | Protein-coding | [rs81000761](https://uswest.ensembl.org/Sus_scrofa/Variation/Explore?db=core;tl=L04JT1jEsfd9sjP1-6853562;v=rs81000761) |

| **SNP** | **Location** | **Allele** | **Consequence** | **Existing variant** |
| --- | --- | --- | --- | --- |
| WU_10.2_6_138715609 | [6:150780123-150780123](https://uswest.ensembl.org/Sus_scrofa/Location/View?contigviewbottom=variation_feature_variation%3Dnormal;db=core;r=6:150780073-150780173;tl=D6mMRoDDSyyney6g-6853565) | A | Intergenic variant | [rs346261987](https://uswest.ensembl.org/Sus_scrofa/Variation/Explore?db=core;tl=D6mMRoDDSyyney6g-6853565;v=rs346261987) |

It has been suggested that the 60K chip may contain too many markers due to high LD in domestic pigs (Badke et al., 2012; Nsengimana et al., 2004; Zhang and Plastow, 2011). It is recommended to use 10% of the markers on the chip (Badke et al., 2012) and a cohort of 1000 animals. Since we cannot increase the sample size, we have conducted the test of the first suggestion. We have randomly selected 1%, 2.5%, 5%, 10%, 15%, 20% of the markers and conducted GWAS, repeating the procedure 100 times. We have compared the results with the GWAS results using the whole chip. Significance assessment of individual SNPs did not change when subsets of SNPs were selected, and the Pearson’s correlation coefficient between logarithms of p-values ranged from 0.963 to 0.995. Therefore, the individual p-values are not getting inflated using the high-density dataset.

**Supp Fig 4. Comparison of rrBLUP and PLINK methods for piglet splay leg phenotype.** Correlation coefficient.


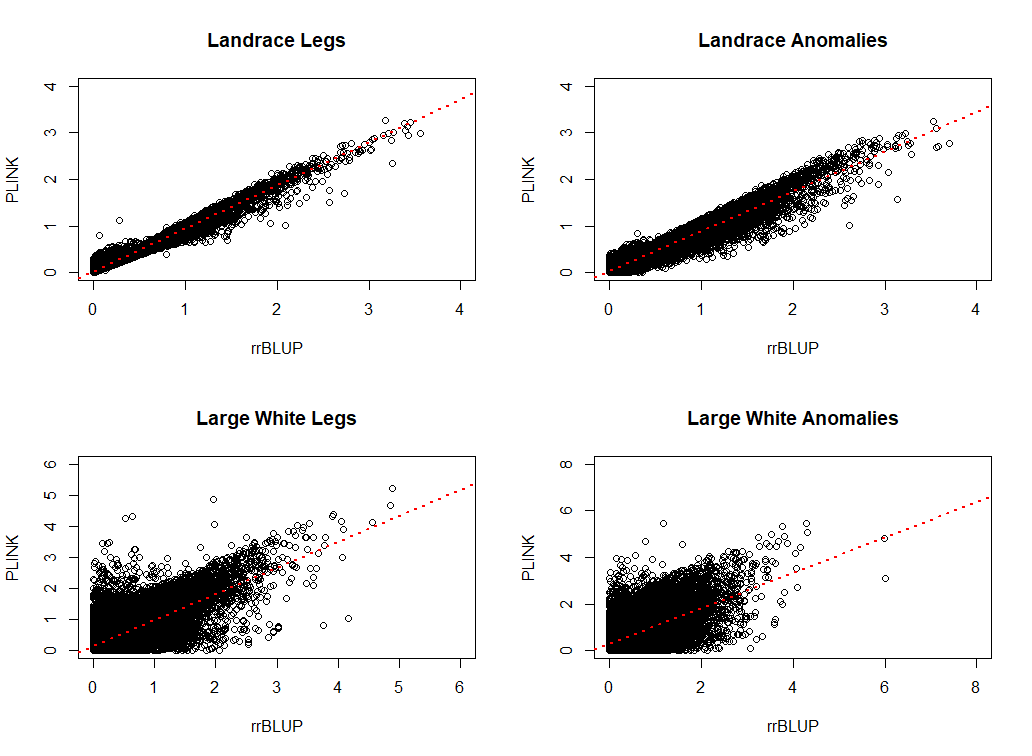


# Supp. Bibliography

Badke, Y.M., Bates, R.O., Ernst, C.W., Schwab, C., and Steibel, J.P. (2012). Estimation of linkage disequilibrium in four US pig breeds. BMC Genomics *13*, 24.

Nsengimana, J., Baret, P., Haley, C.S., and Visscher, P.M. (2004). Linkage disequilibrium in the domesticated pig. Genetics *166*, 1395–1404.

Zhang, C., and Plastow, G. (2011). Genomic Diversity in Pig (Sus scrofa) and its Comparison with Human and other Livestock. Curr. Genomics *12*, 138–146.
